# An Innovative Filtering System for the Handling of Asbestos-Based Products: Improvement of Safety and Quality of Work in Analysis Laboratories

**DOI:** 10.3390/toxics10060281

**Published:** 2022-05-25

**Authors:** Oriana Motta, Concetta Pironti, Marta Venier, Antonio Proto

**Affiliations:** 1 Dipartimento di Medicina, Chirurgia e Odontoiatria “Scuola Medica Salernitana”, University of Salerno, Via S. Allende, 84081 Baronissi, Italy; cpironti@unisa.it; 2 O’Neill School of Public and Environmental Affairs, Indiana University, Bloomington, IN 47405, USA; mvenier@indiana.edu; 3 Dipartimento di Chimica e Biologia, University of Salerno, Via Giovanni Paolo II, 132, 84084 Fisciano, Italy; aproto@unisa.it

**Keywords:** asbestos exposure, analysis laboratory, filter unit removal, work safety

## Abstract

Although being banned or restricted in many countries since the early 1990s, large quantities of asbestos are still used or present in building materials all over the world and its removal or handling requires specific systems that limit exposure to airborne fibers The exposure to asbestos causes chronic diseases such as asbestosis and lung cancer with an incubation period of 20 to 50 years. Among the operators most exposed to contamination are those who handle and analyze the materials in laboratories. For this reason, our work focused on an innovative method for removing a filter unit from a laboratory extraction hood, in order to improve the safety conditions for the operators and the surrounding environment. The hood has a particular construction technology with a mechanism that allows the spraying of a special encapsulating liquid on the ULPA filters below the work-bench, which is capable of forming a film and blocking the fibers on the surface of the same filter. The fibers are irreversibly bounded and can no longer be released into the surrounding environment. The monitoring of activity highlighted the absence of asbestos fibers in the air after installation of the filter and workers feel safer performing their activities. The introduction of an innovative filtering system enhanced the safety of work activities involving asbestos exposure, moreover, the time spent on the hood’s maintenance and the risk perception of workers were improved.

## 1. Introduction

During the 20th century, particularly during and after the second World War, asbestos has been broadly used in many industries and the construction field for its physicochemical properties, such as its flexibility and resistance to traction and heat. Asbestos was also used in walls or around plumbing for insulation functions, to strengthen blocks of cement, or just as a cheap filler. Moreover, asbestos was extensively used for spraying on the underside of the roof in many industrial buildings, and therefore the release of fibers to the surroundings with natural wearing out is common. In a modern city, asbestos can also be found in places such as in the cellar, where steam pipes are insulated, in air conditioning sets, in storage or laundry rooms, in museums, theatres, restaurants, etc. [1,2,3]. When any construction or machine that contains asbestos is damaged, the possibility of exposure is fairly high; in particular, if asbestos is present in a building, it will circulate through all rooms and it is practically impossible to remove. It was designated as a Group 1 carcinogen by the International Agency for Research on Cancer after findings indicated that asbestos could cause lung cancer, malign mesothelioma, asbestosis, and cause fatal damage to the human body [4,5,6,7]. The EU agreed to prohibit the use of asbestos from 1 January 2005, according to regulatory guidelines on asbestos by the Commission Directive 1999/77/EC. However, worldwide asbestos exposure is still a problem. The Global Burden of Disease (GBD) project estimated that 125 million people are exposed to asbestos globally each year [8]. Even if the mineral is no longer produced in most industrialized western countries, the total world production remains high. The mineral has been found in samples of Greenland ice [2] and on the Yorkshire Moors [3], thus indicating worldwide pollution with asbestos that could potentially affect everyone also in non-work settings [9].

However, occupational exposure is the most common form of asbestos exposure. In asbestos extraction and processing sites, workers were the most exposed to the inhalation of thin and long fibers dispersed in the air. Occupational and non-occupational asbestos exposures play a well-known role in determining asbestosis, pulmonary function decline, lung cancer, and mesothelioma [10,11,12,13,14]. A common way of estimating lifelong exposure is the analysis of fibers in the lungs. Although the correlation between lifetime cumulative exposure and fiber concentrations in the lungs is not excellent, in most studies a clear dose–response relationship was established between exposure and the number of fibers in the lungs [15,16,17,18]. Several studies investigated the relationship between workplace asbestos exposure and other type of cancer [19]. Although asbestos damages primarily the airways (oral cavity, pharynx, larynx, and lung), some studies suggested a correlation between asbestos occupational exposure and kidney cancer [20]. In addition to inhalation exposure to asbestos fibers among workers, oral ingestion of the carcinogenic fibers may be associated with gastrointestinal (GI) cancers, especially gastric cancer. Gastric cancer is the fifth most common cancer worldwide and the third-most deadly cancer [21]. Moreover, an association between asbestos exposure and prostate cancer (PCa) has been suggested [22,23]; however, this association does not seem to enhance the incidence of mortality, and workers with occupational asbestos exposure have a PCa incidence and mortality similar to that of the general population [24]. Even though asbestos exposure occurs primarily in the working environment, it is believed that several thousands of deaths can be attributed to non-occupational exposure. The burden of asbestos-related diseases is still rising, even in countries that have banned the use of asbestos in the early 1990s, because of the long latency periods attached to the diseases in question. The incidence of asbestos-related diseases is related to fiber type, fiber size, fiber dose, and the processing operations [25].

In Italy, as in other countries where asbestos-containing materials were totally banned, the occupational health risk could be derived only from exposure from licensed work activities related to asbestos elimination such as asbestos remediation, insulation removal and disposal, or from asbestos products not disposed of properly and that are present in landfills or contaminated places. At the same time, workers also involved in the characterization and chemical–physical analyses of asbestos-containing samples are exposed to the risk of contamination during daily operations in laboratories. There is a great need to improve measures for protecting operators and their surroundings, in particular during critical operations such as the removing and replacing of filter units of laboratory extraction hoods. In Italy, physicians with occupational expertise and the workers’ compensation authority (INAIL) centrally handle the surveillance activities in the workplaces, and collect and store data on exposed workers that are involved in activities entailing the risk of asbestos exposure.

This paper reports on an innovative method for removing a filter unit from a laboratory extraction hood, to improve the safety conditions for the operators and the surrounding environment. The hood has been designed for the safe handling of supports and materials containing asbestos (both massive and in the form of airborne fibers deposited on the filters) as well as potentially toxic dust, ensuring the overall protection of both the operator and the environment not only in the phases of work activities but above all during the maintenance phase in which the change of the ULPA (Ultra Low Penetration Air) filter is performed. This phase is too often underestimated but presents evidence of the presence of critical and dangerous elements.

## 2. Materials and Methods

### 2.1. Structure and Mechanism of the Innovative Filtering System

**Figure 1:**
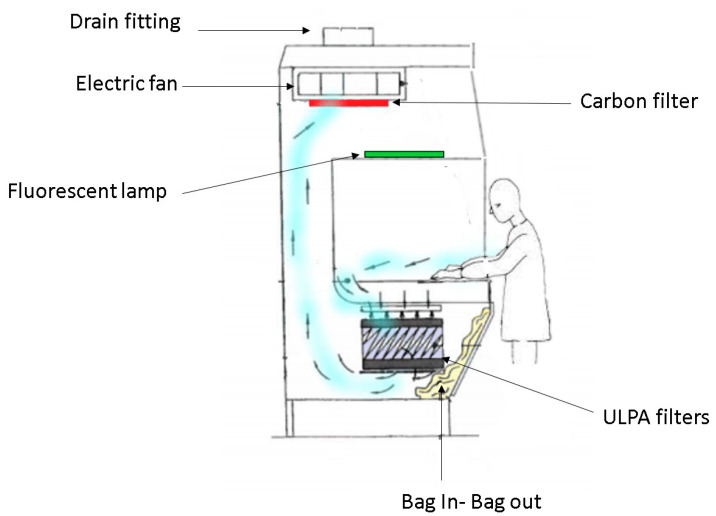
Schematic and section side view of the laboratory extraction hood comprising the filtering and safety removal kit.

**Figure 2:**
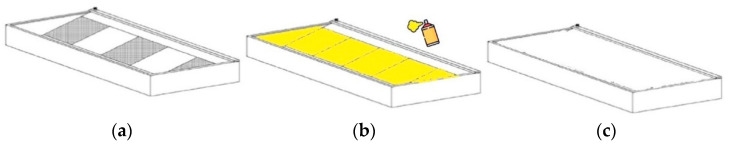
Picture of (**a**) filter before spraying the encapsulating agent; (**b**) colored film capable of blocking the fibers; (**c**) lifting jacks and closed sarcophagus for the safe transportation.

The hood, protected by European patent n. EP 3 093 059 A1 [26], is reported in Figure 1. This new and unique equipment is made by a special internal hydraulic circuit, operated from the outside via a quick coupling located externally on the hood support. This mechanism allows us to spray a special “encapsulating” liquid on the ULPA filters below the work-bench. Before removal of the filter, the unit is automatically sprayed with an encapsulating solution that is able to form a film that captures fibers or powders (see Figure 2). This solution contains a liquid glue that can solidify at room temperature, without requiring the use of organic solvents. The solution is colored as a visual aid to ensure that the entire surface has been coated to enhance operator safety. Once hardened, all the fibers present on the filter are irreversibly bound and can no longer be released into the surrounding environment. Once the hatch has been removed from the access opening, each lamina element is inserted into the suitable guides of the enclosure housing. After the solution has completely solidified—a process that can require from a few minutes to a few hours according to the specific solution used—a solid film is generated on the filter unit. The whole system is then closed with two sliding sheets and can be transported in extreme safety to the disposal site.

### 2.2. Collection, Identification, and Quantification of Inorganic Fibers

Sampling of the inorganic fibers was done by collecting air samples in triplicate with an air volume from 3000 to 3700 L. The obtained three samples were combined into a single representative sample. The sampling point in the laboratories was always the same and near their source.

To quantify asbestos fibers, 10 mg of sampling dust was ground in isopropyl alcohol to obtain a size from 10 to 100 um. After alcohol evaporation, 5 mg of dust was suspended in 200 mL of 0.1% surfactant solution and filtered on a polycarbonate membrane with a 2 mm diameter and a porosity of 0.4–0.8 µm. All membranes prepared for analysis were attached to a glass slide by using acetone vapor (clarification); the filters used for SEM-EDS observation were glued onto the SEM aluminum pin stub by adhesive type. These last filters were also made conductive by carbon sputter coating prior to the SEM-EDS study. The third portion of 500 mg was dehydrated in a drying oven at 60 °C, in order to measure its dry weight, which was used to determine the concentration of fibers. Identification and quantification of inorganic fibers were carried out by SEM-EDS at 2000 magnification and, to minimize both time and costs, by observing a portion of the filter.

The identification and quantification of inorganic fibers were carried out by a Scanning Electron Microscope (SEM, Cambridge Stereoscan S-360 by CAMBRIDGE SCIENTIFIC PRODUCTS 199 Dexter Avenue, Watertown, MA 02472, USA) equipped with an Energy Dispersive Spectrometer (EDS, Link-Oxford Pentafet ATW2, Si(Li) detector by Oxford Instruments, Tubney Woods, Abingdon, UK).

## 3. Results

Replacing a filter unit from a laboratory hood is a very delicate operation that requires extreme caution and suitable measures to protect the operator and the outer environment from possible contamination from hazardous substances that accumulate on the filter unit from the analyzed samples. However, in the presence of highly hazardous contaminated substances, such as the particles or fibers of asbestos or other contaminants and toxic materials, the protective methods adopted by an operator, for example gloves and masks, are often not sufficient to avert the risk of contamination. Suitable nebulized solutions such as formalin or peroxide, which are currently used to sterilize laboratory extraction hoods before removing or replacing filters, suppress possible contaminating microorganisms but have no effects on the previously mentioned asbestos fibers that can expose the operator to a high risk of developing cancer. In this study, we report the use of an especially designed laboratory filter unit that protects the environment and operators during the operations of replacing it.

The effectiveness of using the new filtering system was assessed by periodically monitoring the air quality of the laboratory where the hood containing the new filter was installed and where the measurements are conducted (Table 1).

Based on the data obtained, it is possible to underline that the new filtering system does not allow the unwanted dispersion of asbestos fibers into the surrounding environment, whereas during previous monitoring, before the installation of the new filtering system, asbestos fibers were found in concentrations below 0.1 ff/L, particularly during filter replacement operation.

## 4. Discussion

Asbestos exposure has been a significant source of risk for Italian workers, because of the large use of asbestos and asbestos-containing materials. The Italian Registry of Malignant Mesothelioma (ReNaM) has documented a total of 27,356 reported cases of malignant mesothelioma in Italy from 1993 to 2015 [27].

The removal of contaminated materials releases asbestos fibers into the air with high inhalation hazards both for the workers and for the occupants close to the worksite. The highest exposure levels occur during the re-packaging of asbestos containers, dry cutting of asbestos-containing products, and maintenance of the equipment. The workers use specific personal protective measures (PPE) to protect themselves, such as respiratory equipment, safety goggles, protective gloves, and coveralls; on the other hand, to avoid contamination and the spread of asbestos fibers into the environment the worksite should be under negative pressure. The dispersion of contaminants is related to emission, ventilation rate and air distribution. A recent study evaluated the choice of best practices to guarantee the best control of pressure differences and the air distribution inside an experimental site contaminated by asbestos. The results highlighted that a supply of recirculating air with a differential pressure of at least 10 Pa could be effective for safer work conditions [28].

Bearing in mind that there is no evidence for a threshold for the carcinogenic effect of asbestos, the manipulation of asbestos-containing samples, or even of samples suspected to contain asbestos, should be conducted in the safest possible conditions, ensuring that workers are protected from asbestos exposure. The laboratories that are authorized to analyze asbestos or asbestos-containing materials have to ensure correct safety procedures in the handling of samples within the laboratory and must follow standardized and authorized operating protocols in suitably equipped rooms with decreasing pressure, local exhaust ventilation with filtration, and regular cleaning, as well as including special facilities for the decontamination of the environment. To guarantee maximum personnel safety, the laboratory must provide for a partitioning of the spaces, in order to isolate areas where the handling of asbestos or asbestos-containing samples occur. The laboratory activities related to the manipulation of asbestos-containing materials are to be carried out in suitable rooms equipped with absolute filter aspiration systems (hoods) adopting specific procedures to ensure maximum safety and decontamination actions in case of incidental events. Moreover, periodic environmental monitoring must be carried out for airborne asbestos fibers using both Optical Phase Contrast Microscopy (MOCF), and/or Scanning Electron Microscopy (SEM).

The greatest criticality that can involve a high risk of exposure in a laboratory is the replacement of the filtering group of the hood, as the fibers adhering to the filter can escape from the container box. This phase is too often underestimated and presents evident critical and hazardous elements, also according to Legislative Decree no. 81/08 [29].

In this work, we highlighted the workers’ challenges during the analysis of contaminated materials and the numerous disadvantages of traditional systems during the process of replacement and regeneration of hood filters. We therefore proposed the use of an innovative filtering unit enclosed in a specifically designed hood where the filter is sprayed with an “encapsulating” liquid solution, which forms a film that is capable of blocking the fibers on the surface of the same filter before being removed. Once the solution is hardened, all the fibers present on the filter are irreversibly bound and can no longer be released into the surrounding environment. In the hood, an internal electric aspirator forces the air to pass first through the ULPA filters, to retain the hazardous substances, and then it is sent to the final HEPA filter to be evacuated. Through the integration of the hydraulic circuit into the hood structure, and its operation from the outside, the operator can avoid any kind of contact with the internal parts (worktop, container filters, panels, etc.). The filtering group is contained in a special box equipped with two lifting jacks in order to position the unit to match the inclined frame placed under the worktop, guaranteeing a dynamic sealing and therefore ensuring that no material, both during the work phases and during the operation of changing filters, can escape from the bin that contains them.

In the literature, a very recent study reported the development of a mechanistic model (Asbestos Removal Exposure Assessment Tool (AREAT)) to estimate asbestos fiber exposure released during asbestos abatement processes, which is equipped with a library of safe working procedures specific for each environment [30]. Models are a simplification and never a perfect representation of reality; therefore, cautious interpretation is needed, however they have been shown to be very helpful since it is a costly and time-consuming process to measure all situations, as working conditions and materials may vary greatly. This innovative system can be considered to be an innovative approach to use in control strategies to avoid exposure during working procedures, leading to a zero emission potential and exposure risk.

The results of the present study showed that the use of the solid solution improves work safety conditions, ultimately reducing occupational exposure to asbestos in laboratory settings. All the operators who have used this system feel better protected and carry out their functions with an increased peace of mind. Within the many actions proposed to enhance the security levels in managing asbestos-containing materials, this is a low-cost and easy method with respect to other solutions presented in the literature, such as the mobilization of chrysotile asbestos fibers on natural zeolite substrate, clinoptilolite, geopolimers [31], and vetrification processes [32,33].

All the laboratories that used this system confirmed the total absence of asbestos fibers and airborne mineral fibers in the work environment following personal monitoring of exposed operators and environmental analyzes by SEM and/or MOCF, according to the analytical methods recommended by the specific legislation in force (DM 6/9/94, Legislative Decree 81/08). Analysts felt fully satisfied and better protected in the workplace while handling asbestos-containing materials.

## 5. Conclusions

The fundamental safety requirement concerning the “asbestos risk” in workplaces handling asbestos containing products or samples is fully satisfied with this proposed new system. The operators carry out their functions efficiently while also being protected during all crucial steps in the handling of samples that lead to a possible release of fibers into the environment. The solid film permanently fixes all the particles and fibers to the filter unit, and in general, any possible harmful substance coming from the analyzed sample present on the filter unit, preventing their release into the environment. Moreover, this system presents further advantages when compared to other systems such as lower noise; an easy removal of the filter, and a shorter time required for its replacement; reduction of laboratory interruption for replacement and maintenance operations; greater safety for operators and assistance staff. This could be considered as an implementation of the measures to prevent asbestos exposure in place during filter removal, as requested by Italian law (Legislative decree 81/08). Article 225 of Legislative Decree 81/08, regarding specific protection and prevention measures, establishes that the employer, on the basis of the activity and risk assessment, is required to ensure that the risk is either eliminated or reduced by replacing it with other agents or processes which, in the conditions of use, are not dangerous or are less hazardous for the health of workers.

The traditional processes used in scientific laboratories involve time-consuming and complicated procedures for replacing hood filters with high costs and interruptions to normal operations. In our study, all laboratories involved described the new methodologies as safe, convenient, and easy to use.

## 6. Patents

EP 3 093 059 A1 Method for removing a filter unit from a laboratory extraction hood, relative filtering and safety removal kit, and laboratory extraction hood. https://patentimages.storage.googleapis.com/e6/c7/08/ad0e7ecc0538cf/EP3093059A1.pdf. (accessed on 20 April 2022).

## Figures and Tables

**Table 1:** Evaluation of environmental ambient air asbestos concentration.

Sample	Date	Volume (L)	Fibers (n)
1	16 July 2019	3368	0
2	16 January 2020	3000	0
3	24 January 2020	3000	0
4	25 January 2020	3000	0
5	29 January 2020	3000	0
6	30 January 2020	3000	0
7	22 September 2020	3707	0
8	16 July 2021	3300	0

## Data Availability

Not applicable.
